# Effect of High-Frequency Stimulation of the Perforant Path on Previously Acquired Spatial Memory in Rats: Influence of Memory Strength and Reactivation

**DOI:** 10.1371/journal.pone.0100766

**Published:** 2014-06-27

**Authors:** Katherine G. Akers, Derek A. Hamilton

**Affiliations:** 1 Department of Psychology, University of New Mexico, Albuquerque, New Mexico, United States of America; 2 Department of Neurosciences, University of New Mexico, Albuquerque, New Mexico, United States of America; Centre national de la recherche scientifique, University of Bordeaux, France

## Abstract

If memory depends on changes in synaptic strength, then manipulation of synaptic strength after learning should alter memory for what was learned. Here, we examined whether high frequency stimulation of the perforant path *in vivo* disrupts memory for a previously-learned hidden platform location in the Morris water task as well as whether this effect is modulated by memory strength or memory reactivation. We found that high frequency stimulation affected probe test performance regardless of memory strength or state of memory activation, although the precise nature of this effect differed depending on whether rats received minimal or extensive training prior to high frequency stimulation. These findings suggest that artificial manipulation of synaptic strength between the entorhinal cortex and hippocampus may destabilize memory for a previously-learned spatial location.

## Introduction

Connectionist models propose that memories are represented by specific patterns of changes in synaptic strength across a neural network [1], [2]. If so, then post-learning manipulation of synaptic strength within the network should alter memory for what was learned [2], [3]. Indeed, studies report that high-frequency stimulation (HFS) of perforant path-dentate gyrus synapses after training in the Morris water task [4] or a dry-land maze [5] results in retrograde amnesia for the spatial memory. Presumably, the artificial, HFS-induced increase in synaptic strength interferes with natural, learning-induced changes in synaptic strength, resulting in degradation of the memory trace.

In initial work seeking to determine the conditions under which manipulation of synaptic strength alters memory, it was found that weak spatial memories (i.e., those produced by minimal training) were disrupted by perforant path HFS, but strong spatial memories (i.e., those produced by extensive training) were unaffected [5]. In addition to memory strength, another factor that may influence whether manipulation of synaptic strength alters memory is the state of memory activation, as the return of a memory to an active state can render that memory more vulnerable to amnestic agents [6], [7]. Here, we examine (1) whether reactivation of memory for the location of a hidden platform in the water task renders the memory more vulnerable to disruption by perforant path HFS and (2) whether the effect of memory reactivation differs for weak versus strong memories. We found that HFS affected behavioral performance in the water task regardless of the state of memory activation or memory strength, although the precise nature of this effect differed depending on whether rats received minimal or extensive training prior to HFS.

## Materials and Methods

### Ethics statement

All procedures were approved by the Institutional Animal Care and Use Committee at the University of New Mexico (protocol 06MCC021) and conformed to the National Research Council's Guide for the Care and Use of Laboratory Animals.

### Rats

**Figure 1:**
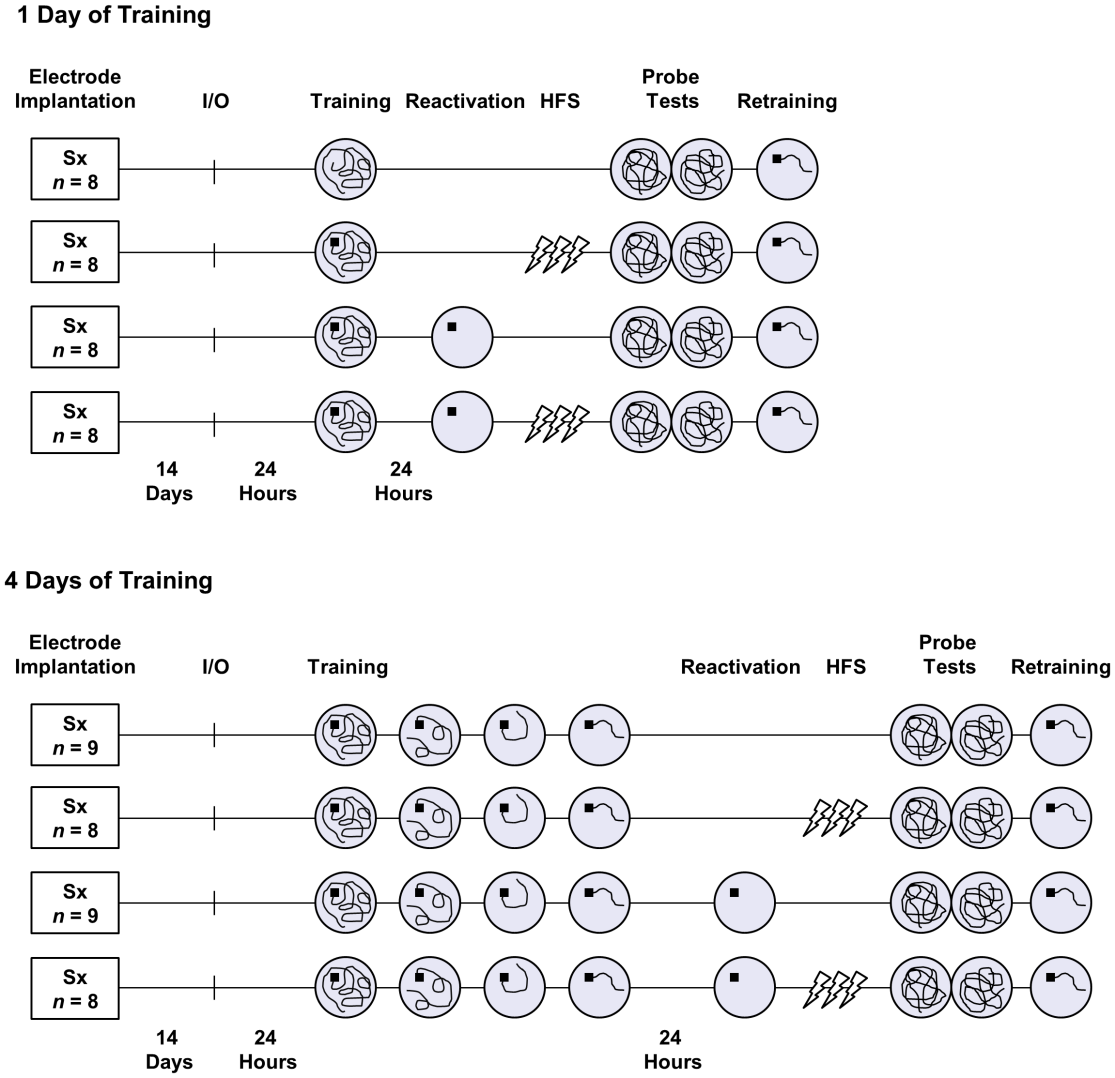
Experimental design and timeline. Rats were surgically (Sx) implanted with stimulating electrodes in the perforant path and recording electrodes in the dentate gyrus. After the acquisition of I/O curves, rats underwent either 1 day (upper timelines) or 4 consecutive days (lower timelines) of training in the hidden platform version of the Morris water task. Twenty-four hours after the completion of training, half of the rats received a memory reactivation treatment consisting of being briefly placed on the platform in its trained location in the pool (reactivation group), whereas the other half remained in their home cages (no reactivation group). Next, half of the rats received 10 HFS trains applied bilaterally to the perforant path (HFS group), whereas the other half received a matching number of test pulses (control group). Afterward, rats' memory for the platform location was assessed during two consecutive probe tests with the platform removed from the pool. Finally, rats underwent retraining with the platform returned to its trained location.

Adult (3–5 months old) male Long-Evans rats were obtained from Harlan (Indianapolis, IN) or bred at the Psychology Department animal research facility (from Harlan stock). Rats were housed individually on a 12-hr light/dark cycle with food and water available ad libitum. All procedures and behavioral testing occurred during the light phase. Experimental timelines are shown in Figure 1.

### Surgery

Under isoflurane anesthesia, two recording electrodes and two stimulating electrodes (Teflon-coated stainless steel wires, 114 µm outer diameter) were surgically implanted under stereotaxic guidance. Recording electrodes were placed bilaterally in the hilar region of the dentate gyrus (−3.5 mm AP, ±1.8 mm LV, and 3.6–4.6 mm DV relative to bregma). Stimulating electrodes were placed bilaterally in the medial perforant path (−8.1 mm AP, ±4.3 mm LV, and 4.0–5.0 mm DV relative to bregma). Electrodes were lowered until a positive excitatory postsynaptic potential (EPSP) with a superimposed negative population spike (PS) was evoked by test pulses (100 µs duration, 10 s inter-pulse-interval, 300 µA). Five stainless steel jewelers screws were tapped into the skull; two served as reference and ground components of the differential recording circuit, one served as a return component for the stimulating circuit, and two provided additional structural support. Electrodes were fixed in place with dental cement and anchored to the skull using the skull screws. Following surgery, rats were given 14 days to recover before the onset of behavioral procedures.

### Input/output (I/O) curves

To habituate rats to the stimulating/recording procedures, rats were transported in their home cages to the electrophysiology room, where they remained for ∼30 min and were handled by the experimenter for ∼5 min. Rats received 3 such habituation sessions on separate days. During the final habituation session, I/O curves were acquired from each hemisphere by applying 20 test pulses (100 µs duration, 10 s inter-pulse-interval) at 3 current intensities: 100, 300, and 500 µA. Evoked response size was measured by the slope of the initial rising phase of the EPSP and the amplitude of the superimposed negative PS.

### Training

One day after acquisition of I/O curves, rats underwent training in the Morris water task. A circular pool, 1.5 m in diameter and 48 cm in height, was filled to a depth of 26 cm with water made opaque by the addition of non-toxic powdered paint. The temperature of the water was ∼22°C. An escape platform (15 cm×15 cm) was hidden ∼1 cm below the surface of the water and remained in a fixed position throughout training. Rats received either 1 or 4 consecutive days of training, with 12 trials per day. Trials began when rats were released from one of four points equally spaced around the perimeter of the pool with their heads facing the pool wall. Each release point was used an equal number of times per day, and the sequence of release points was randomized within and across days. Trials ended when rats reached the platform. If a rat did not reach the platform within 60 s, the trial was terminated and the rat was placed on the platform by the experimenter. Rats were removed from the platform after ∼5 s and placed in holding cages between trials. The inter-trial interval was ∼5 min. Behavior was videotaped via an overhead camera and digital camcorder. The digital video was transferred to a Linux workstation for tracking and analysis of swim paths using custom software developed in our laboratory. Performance during training was measured by latency to reach the platform.

### Memory reactivation

One day after the completion of training, rats in the reactivation group were given a memory reactivation treatment, which consisted of being placed on the submerged platform in its trained location in the pool for 30 s. Rats in the no reactivation group remained in their home cages in the colony room during this period of time.

### High-frequency stimulation (HFS)

Immediately after the reactivation treatment, rats were transported to the electrophysiology room. First, baseline evoked responses were recorded (20 pulses, 100 µs duration, 10 s inter-pulse-interval, 300 µA). Next, rats in the HFS group received 10 HFS trains (10 pulses/train delivered at 400 Hz, 100 µs duration pulses, 30 s inter-train interval, 400 µA), and rats in the control group received an equal number of test pulses (100 pulses, 100 µs duration, 10 s inter-pulse-interval, 400 µA) to match the total amount and intensity of current received by rats in the HFS group. Finally, post-HFS evoked responses were recorded (20 pulses, 100 µs duration, 10 s inter-pulse-interval, 300 µA). This sequence was completed for one hemisphere at a time in succession, with order (left-right or right-left) counterbalanced within each experimental condition. HFS-induced potentiation of evoked responses was measured by the percentage increase in EPSP slope and PS amplitude above pre-HFS levels (i.e., (post-HFS/pre-HFS ×100) −100). Rats in the HFS and control groups were handled similarly throughout the stimulating/recording procedures and remained in the electrophysiology room for an equivalent length of time (∼1 hour).

### Probe tests and retraining

After HFS or a matching number of test pulses, rats' memory for the platform location was assessed during two consecutive 60-s probe tests during which the platform was removed from the pool. The use of two consecutive probe tests as opposed to a single probe test was based on previous findings that differences between groups in memory for the platform location may not be apparent during the first probe test but may emerge during subsequent probe test [8], [9]. Rats were released from one of two release points located far from the former platform location. Each release point was used once for each rat, and the sequence of release points was counterbalanced within experimental conditions. The two probe tests were separated by a 1-min interval, during which rats were placed in holding cages. Performance during the probe tests was assessed using six dependent measures: (1) latency and (2) length of path to the target location (i.e., former platform location), which are measures of initial trajectory, and (3) number of target location crosses, (4) time spent in a circular zone (25-cm diameter) centered on the target location, and (5) time spent in the quadrant containing the target location, which are measures of persistence in searching, and (6) swim speed.

After the second probe test, rats were returned to the holding cages for 1 min. The platform was then returned to the target location, and rats were given 8 retraining trials using an identical procedure as that used during initial training. Performance during retraining was measured by latency to reach the platform.

### Statistical analysis

I/O and behavioral data were initially analyzed using omnibus analysis of variance (ANOVA) with HFS, reactivation, and training as between-subject factors, and current (for I/O data), trial (for training and retraining data), or probe (for probe test data) as within-subject factors. Significant interactions were followed by separate ANOVAs within specific factors of interest. Changes in EPSP slope and PS amplitude following HFS or a matching number of test pulses were analyzed using one-sample *t*-tests. Planned comparisons were performed to test whether rats that received 4 days of training performed better than rats that received 1 day of training at the end of training.

## Results

### I/O curves

**Figure 2:**
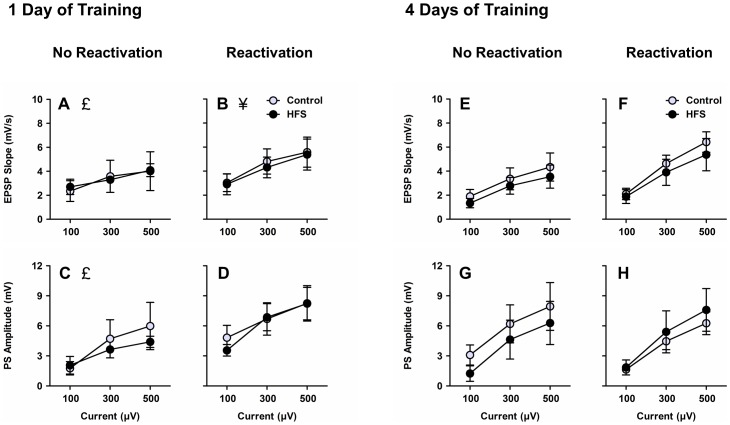
I/O curves. Across all experimental conditions, increasing intensities of current applied to the perforant path led to reliable increases in the size of evoked responses in the dentate gyrus considering both EPSP slope (**ABEF**) and PS amplitude (**CDGH**). £ denotes main effect of current (collapsed across 1-day/4-day training, reactivation/no reactivation, and HFS/control groups), *p*<.05. ¥ denotes reactivation × current interaction (collapsed across 1-day/4-day training and HFS/control groups), *p*<.05.

Naive rats were surgically implanted with bilateral stimulating electrodes in the perforant path and bilateral recording electrodes in the dentate gyrus region of the hippocampus. After recovery from surgery, I/O curves were obtained. Across all experimental conditions, increasing intensities of current applied to the perforant path reliably led to increases in the size of responses in the dentate gyrus, considering both EPSP slope (Figure 2ABEF, current main effect, *F*_2,100_ = 62.60, *p*<.001) and PS amplitude (Figure 2CDGH, current main effect, *F*_2,100_ = 53.55, *p*<.001). Rats assigned to the reactivation group exhibited larger EPSP slope at the highest current intensity compared to rats assigned to the no reactivation group (reactivation × current interaction, *F*_2,100_ = 3.91, *p* = .023; 500 µA: *t*_56_ = 2.03, *p* = .047), but there was no such difference between groups considering PS amplitude (reactivation × current interaction, *p*>.05). There were no significant effects of training or HFS group assignment on EPSP slope or PS amplitude (*p*s>.05).

### Training

**Figure 3:**
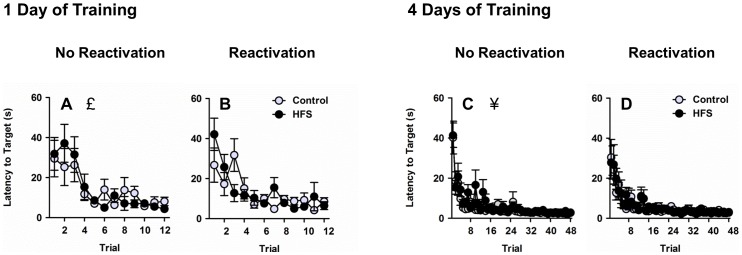
Training. Both rats that underwent 1 day of training (**AB**) and rats that underwent 4 days of training (**CD**) displayed a decrease in latency to reach the hidden platform across trials. £ denotes main effect of trial within 1-day training group (collapsed across reactivation/no reactivation and HFS/control groups), *p*<.05. ¥ denotes main effect of trial within 4-day training group (collapsed across reactivation/no reactivation and HFS/control groups), *p*<.05.

Rats received either 1 or 4 days of training in the Morris water task, with 12 training trials per day. Throughout training, the hidden platform remained in a fixed spatial location relative to distal cues. A decrease in latency to reach the platform across training trials was observed for rats that received 1 day of training (Figure 3AB, trial main effect within the 1-day training group, *F*_11,308_ = 15.24, *p*<.001) and rats that received 4 days of training (Figure 3CD, trial main effect within the 4-day training group, *F*_47,1410_ = 22.93, *p*<.001). Planned comparisons confirmed that rats that received 4 days of training achieved superior levels of performance compared with rats that received 1 day of training, evidenced by shorter average latencies to reach the platform during the final four training trials (trials 9–12 for the 1-day training group vs. trials 45-48 for the 4-day training group, training main effect, *F*_1,83_ = 35.96, *p*<.001). There were no significant effects of reactivation or HFS group assignment on training performance (*p*s>.05).

### HFS

**Figure 4:**
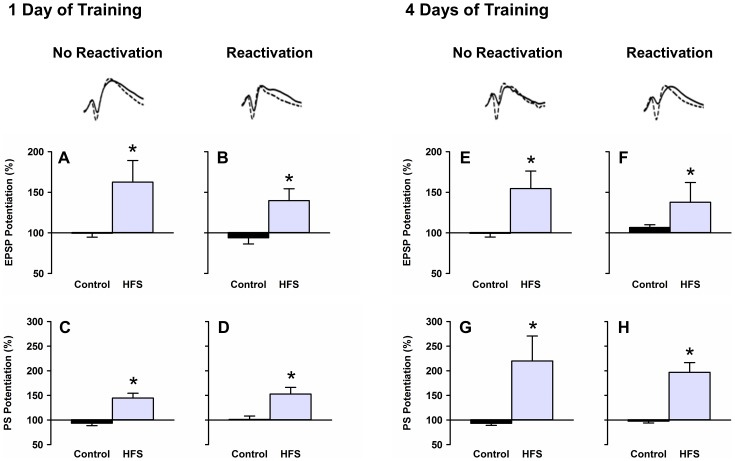
HFS. Across all experimental conditions, rats in the HFS group exhibited HFS-induced potentiation of evoked responses considering both EPSP slope (**ABEF**) and PS amplitude (**CDGH**), whereas rats in the control group exhibited no change in the size of evoked responses after a matching number of test pulses. Above graphs: representative traces of evoked responses from rats in the HFS group before (solid line) and after (dashed line) HFS. * denotes HFS-induced potentiation of evoked responses above baseline, *p*<.05.

Twenty four hours after the completion of training, rats in the reactivation group were placed on the hidden platform at its trained location in the pool for 30 s, while rats in the no reactivation group remained in their home cages. Immediately afterward, rats in the HFS group received 3 trains of HFS applied bilaterally to the perforant path, and rats in the control group received a matching number of test pulses. Across all experimental conditions, rats in the HFS group exhibited significant potentiation of dentate gyrus evoked responses relative to baseline considering both EPSP slope (Figure 4ABEF, 1-day training reactivation: *t*_7_ = 2.73, *p* = .029; 1-day training no reactivation: *t*_5_ = 3.19, *p* = .024; 4-day training reactivation: *t*_7_ = 2.68, *p* = .037; 4-day training no reactivation: *t*_6_ = 2.54, *p* = .029) and PS amplitude (Figure 4CDGH, 1-day training reactivation: *t*_7_ = 3.91, *p* = .006; 1-day training no reactivation: *t*_5_ = 4.64, *p* = .006; 4-day training reactivation: *t*_7_ = 4.88, *p* = .037; 4-day training no reactivation: *t*_6_ = 2.75, *p* = .033), whereas rats in the control groups exhibited no change in the size of evoked responses (*p*s>.05).

### Probe tests

Approximately 20 minutes after completion of HFS or a matching number of test pulses, rats received two consecutive probe tests in the water task with the platform removed from the pool. Omnibus ANOVAs revealed several significant higher-order interactions on latency to the target location (training × reactivation × probe test interaction, *F*_1,58_ = 5.13, *p* = .027; HFS × probe test interaction, *F*_1,58_ = 4.82, *p* = .032), path length to the target location (training × reactivation × probe test interaction, *F*_1,58_ = 4.59, *p* = .036; HFS × probe test interaction, *F*_1,58_ = 6.39, *p* = .014), number of target crosses (training × probe test interaction, *F*_1,58_ = 5.40, *p* = .024), time in target zone (training × reactivation interaction, *F*_1,58_ = 5.22, *p* = .026; training × probe test interaction, *F*_1,58_ = 7.48, *p* = .008), and time in target quadrant (training × reactivation interaction, *F*_1,58_ = 9.53, *p* = .003). There were significant main effects of training on all five dependent measures (latency, *F*_1,58_ = 11.40, *p* = .001; path length, *F*_1,58_ = 14.97, *p*<.001; crosses, *F*_1,58_ = 11.76, *p* = .001; time in zone, *F*_1,58_ = 23.87, *p*<.001; time in quadrant, *F*_1,58_ = 30.85, *p*<.001), indicating that rats that received 4 days of training took shorter initial trajectories to the target location and persisted longer in searching around the target location compared with rats that received 1 day of training. There were also significant main effects of probe test on all five dependent measures (latency, *F*_1,58_ = 13.31, *p* = .001; path length, *F*_1,58_ = 7.15, *p* = .010; crosses, *F*_1,58_ = 18.51, *p*<.001; time in zone, *F*_1,58_ = 7.25, *p* = .009; time in quadrant, *F*_1,58_ = 6.27, *p* = .015), indicating that rats took longer initial trajectories and persisted less in searching around the target location during the second probe test. Finally, there was a significant main effect of HFS on time in zone (*F*_1,58_ = 4.90, *p* = .031), indicating that HFS rats spent less time in the target zone than control rats. To further analyze differences among experimental conditions, separate ANOVAs were performed within the 1-day and 4-day training groups.

**Figure 5:**
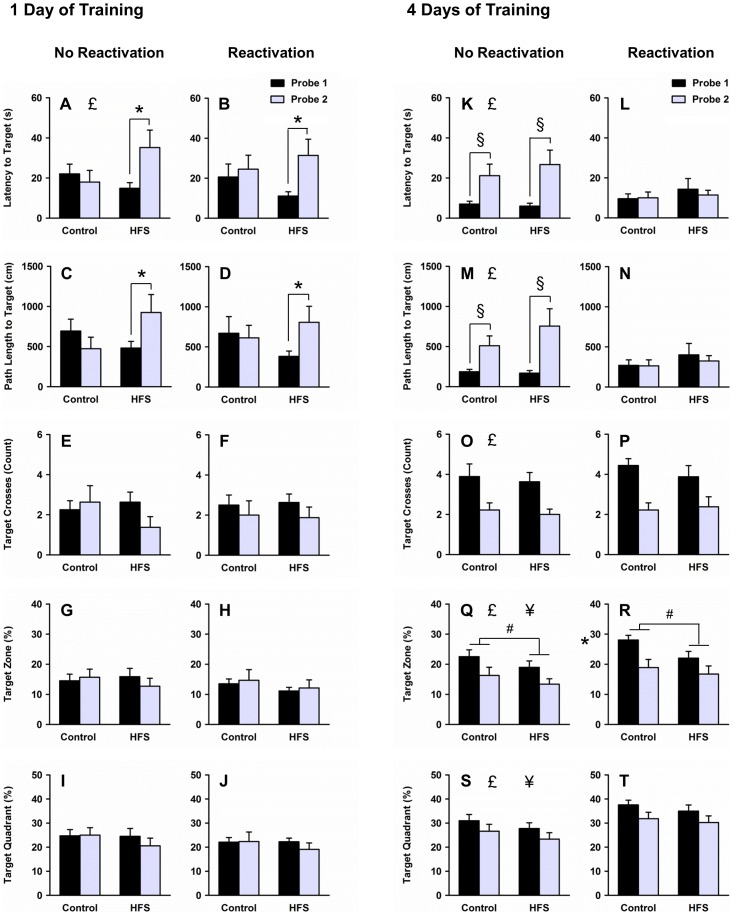
Probe tests. Among rats that underwent 1 day of training, rats in the HFS group exhibited a significant increase in latency (**AB**) and path length (**CD**) to the target location across consecutive probe tests, whereas rats in the control group exhibited no change across probe tests. HFS did not affect number of target crosses (**EF**), time spent in the target zone (**GH**), or time spent in the target quadrant (**IJ**). Among rats that underwent 4 days of training, HFS rats spent less time in the target zone than control rats during both probe tests (**QR**). Also, rats in the reactivation group exhibited short latency (**L**) and path length (**N**) during both probe tests, whereas rats in the no reactivation group exhibited a significant increase in latency (**K**) and path length (**M**) across consecutive probe tests. Rats in the reactivation group also spent more time in the target zone and target quadrant during both probe tests (**RT**) compared to rats in the no reactivation group (**QS**). Neither HFS nor reactivation affected number of target crosses (**OP**). * denotes main effect of probe test within HFS group (collapsed across reactivation/no reactivation groups), *p*<.05.). # denotes main effect of HFS (collapsed across reactivation/no reactivation groups and probe tests), *p*<.05. £ denotes main effect of probe test (collapsed across HFS/control and reactivation/no reactivation groups), *p*<.05. § denotes main effect of probe test within no reactivation group (collapsed across HFS/control groups), *p*<.05. ¥ denotes main effect of reactivation (collapsed across HFS/control groups and probe tests), *p*<.05.

Among rats that received 1 day of training, HFS had a probe test-dependent effect on initial trajectory to the target location (Figure 5ABCD, HFS × probe interaction, latency: *F*_1,28_ = 6.18, *p* = .019, path length: *F*_1,28_ = 6.83, *p* = .014). Across consecutive probe tests, control rats displayed no change in latency or path length to the target location (probe main effects within control group, *p*s>.05). However, HFS rats exhibited a significant increase in latency and path length across consecutive probe tests regardless of whether memory for the platform location was reactivated prior to HFS (probe main effect within HFS group, latency: *F*_1,14_ = 11.91, *p* = .004, path length: *F*_1,14_ = 7.66, *p* = .015). Although HFS rats exhibited shorter latency and path length than control rats during the first probe test and longer latency and path length than control rats during the second probe test, these differences did not reach significance (HFS main effects within probe test 1, *p*s>.05; HFS main effects within probe test 2, *p*s>.05). Across all experimental conditions (i.e., collapsed across HFS/control and reactivation/no reactivation groups), rats exhibited longer latencies to the target location during the second probe test compared to the first probe test (overall probe main effect, *F*_1,28_ = 6.09, *p* = .020). In contrast to measures of initial trajectory, measures of persistence in searching around the target location were not affected by either HFS or reactivation (Figure 5EFGHIJ, *p*s>.05). Furthermore, HFS did not affect swim speed during the first probe test (Text S1; Figure S1AB).

Among rats that received 4 days of training, HFS rats spent less time in the target zone than control rats across both probe tests (Figure 5QR, HFS main effect, *F*_1,30_ = 4.74, *p* = .038). Further analyses revealed that this difference between groups was particularly apparent during the initial 15 s of the probe tests (Text S1; Figure S1MN). There was also a probe test-dependent effect of reactivation on initial trajectory to the target location (Figure 5KLMN, reactivation × probe interaction, latency: *F*_1,30_ = 10.04, *p* = .004, path length: *F*_1,30_ = 10.46, *p* = .004). Whereas rats in the no reactivation group exhibited a significant increase in latency and path length to the target location across consecutive probe tests (probe main effect within no reactivation group, latency: *F*_1,16_ = 13.03, *p* = .002, path length: *F*_1,16_ = 12.91, *p* = .002), rats in the reactivation group displayed short latencies and path lengths across both probe tests (probe main effects within reactivation group, *p*s>.05). Furthermore, across both probe tests, rats in the reactivation group spent more time in the target zone (Figure 5QR, reactivation main effect, *F*_1,30_ = 4.68, *p* = .039) and the target quadrant than rats in the no reactivation group (Figure 5ST, reactivation main effect, *F*_1,30_ = 12.25, *p* = .001). Finally, across all experimental conditions (i.e., collapsed across HFS/control and reactivation/no reactivation groups), rats exhibited longer initial trajectories to the target location and less persistence in searching around the target location during the second probe test compared to the first probe test (probe main effect, latency: *F*_1,30_ = 7.61, *p* = .010, path length: *F*_1,30_ = 7.30, *p* = .011, crosses: *F*_1,30_ = 30.81, *p*<.001, time in zone: *F*_1,30_ = 17.12, *p*<.001, time in quadrant: *F*_1,30_ = 7.52, *p* = .010).

### Retraining

**Figure 6:**
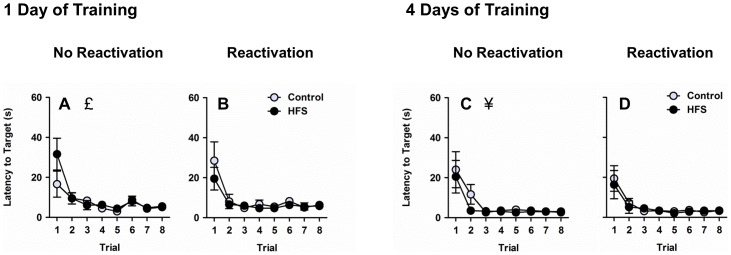
Retraining. Both rats that underwent 1 day of training (**AB**) and rats that underwent 4 days of training (**CD**) displayed a decrease in latency to reach the hidden platform across trials. Rats that underwent 4 days of training exhibited shorter latency than rats that underwent 1 day of training. £ denotes main effect of trial (collapsed across 1-day/4-day, reactivation/no reactivation, and HFS/control groups), *p*<.05. ¥ denotes main effect of training (collapsed across reactivation/no reactivation and HFS/control groups and trial), *p*<.05.

After the probe tests, rats received 8 retraining trials with the platform returned to its trained location in the pool. Rats that underwent 4 days of training reached the platform faster than rats that underwent 1 day of training (Figure 6ABCD, training main effect, *F*_1,58_ = 8.34, *p* = .005). Across all experimental conditions, latency to reach the platform decreased across retraining trials (trial main effect, *F*_7,406_ = 35.49, *p*<.001). Neither HFS nor reactivation had a significant effect on performance during retraining (*p*s>.05).

## Discussion

We found that HFS of the perforant path after rats learned to locate a hidden platform in the Morris water task affected their performance during memory retention testing regardless of memory strength or whether memory for the platform location was reactivated before HFS. Considering rats that were minimally trained (i.e., 1 day), rats that received perforant path HFS showed an increase in latency to reach the former platform location across probe tests, whereas control rats showed no change in latency across probe tests. Considering rats that were extensively trained (i.e., 4 days), rats that received HFS spent less time in a target zone centered on the former platform location compared with control rats across both probe tests.

These findings suggest that perforant path HFS weakened memory for the platform location, although the precise nature of the effect of HFS on probe test performance differed depending on the amount of training rats received. Extensively trained HFS rats spent less time searching for the platform at its former location than control rats across both probe tests. The same effect of HFS was not observed among minimally trained rats, however, perhaps due to a floor effect resulting from both control and HFS rats showing little persistence in searching for the platform at its former location. Rather, minimally trained rats showed a probe test-dependent effect of HFS on measures of initial trajectory to the platform. That is, minimally trained HFS rats reached the former platform location as quickly as control rats during the initial probe test, suggesting that HFS-induced destabilization of memory for the platform location was insufficient to impair performance, perhaps due to pattern completion of fragmentary information transmitted from the dentate gyrus to downstream hippocampal subregions [10], [11]. When the platform was not encountered at its expected location during the first probe test, however, a somewhat destabilized memory may have become further undermined, resulting in a failure to navigate to the former platform location during the second probe test. The possibility that perforant path HFS and the absence of the platform at its expected location could jointly destabilize memory for the platform location is consistent with reports that both HFS of the ventral hippocampal commissure [12] and the dislocation of an escape platform in the water task [13] alter established hippocampal place fields.

Interestingly, this pattern of probe test performance among minimally trained rats could further suggest that the destabilization of previously acquired memory facilitates new learning. Previous studies demonstrate that successive no-platform probe tests after training in the Morris water task result in extinction of spatial preference for the platform location, which is indicative of new learning that the platform is no longer in its original location rather than unlearning of the original platform location [14], [15]. Thus, an HFS-induced increase in latency to reach the former platform location across consecutive probe tests may reflect an acceleration of learning that the platform was no longer in its trained location. This is consistent with the finding in rabbits that perforant path HFS increased the rate of nictitating membrane response conditioning [16]. Thus, instead of administering a retraining session with the platform returned to its original location, future experiments could utilize a new platform location during retraining to test whether HFS rats can form a new spatial memory more quickly than control rats. We also note that rats in the no reactivation group that received extensive training exhibited a similar increase in latency to reach the former platform location across probe tests, which likely reflects accelerated extinction due to continuous reinforcement [17] experienced throughout the extensive training period.

The primary goal of the present study was to determine whether the effect of perforant path HFS on spatial memory depends on two factors: memory strength and the state of memory activation. Considering memory strength, we found that HFS affected probe test performance among both minimally trained and extensively trained rats, although the precise nature of HFS effect differed depending on the amount of training. Considering the state of memory activation, we found that HFS affected probe test performance in similar ways regardless of whether memory for the platform location was reactivated immediately before HFS. Therefore, perforant path HFS may affect memory for a previously learned spatial location regardless of memory strength or state of memory activation, perhaps via artificially inducing changes in the specific pattern of synaptic strengths representing the spatial memory.

## Supporting Information

Figure S1 **Time bin analysis of probe test performance.** (**ABIJ**) Swim speed across 15-s time bins. (**CDKL**) Number of target crosses across 15-s time bins. (**EFMN**) Time spent in a 25-cm circular zone centered on the target location across 15-s time bins. (**GHOP**). Time spent in the target location quadrant across 15-s time bins.
(TIF)

Text S1 **Results of time bin analysis of probe test performance.**
(DOCX)
