# Supplementary figures and images for: Effect of High-Frequency Stimulation of the Perforant Path on Previously Acquired Spatial Memory in Rats: Influence of Memory Strength and Reactivation
Source: PLoS One. 2014 Jun 27;9(6):e100766. doi: 10.1371/journal.pone.0100766 (PMC4074056; doi:10.1371/journal.pone.0100766)

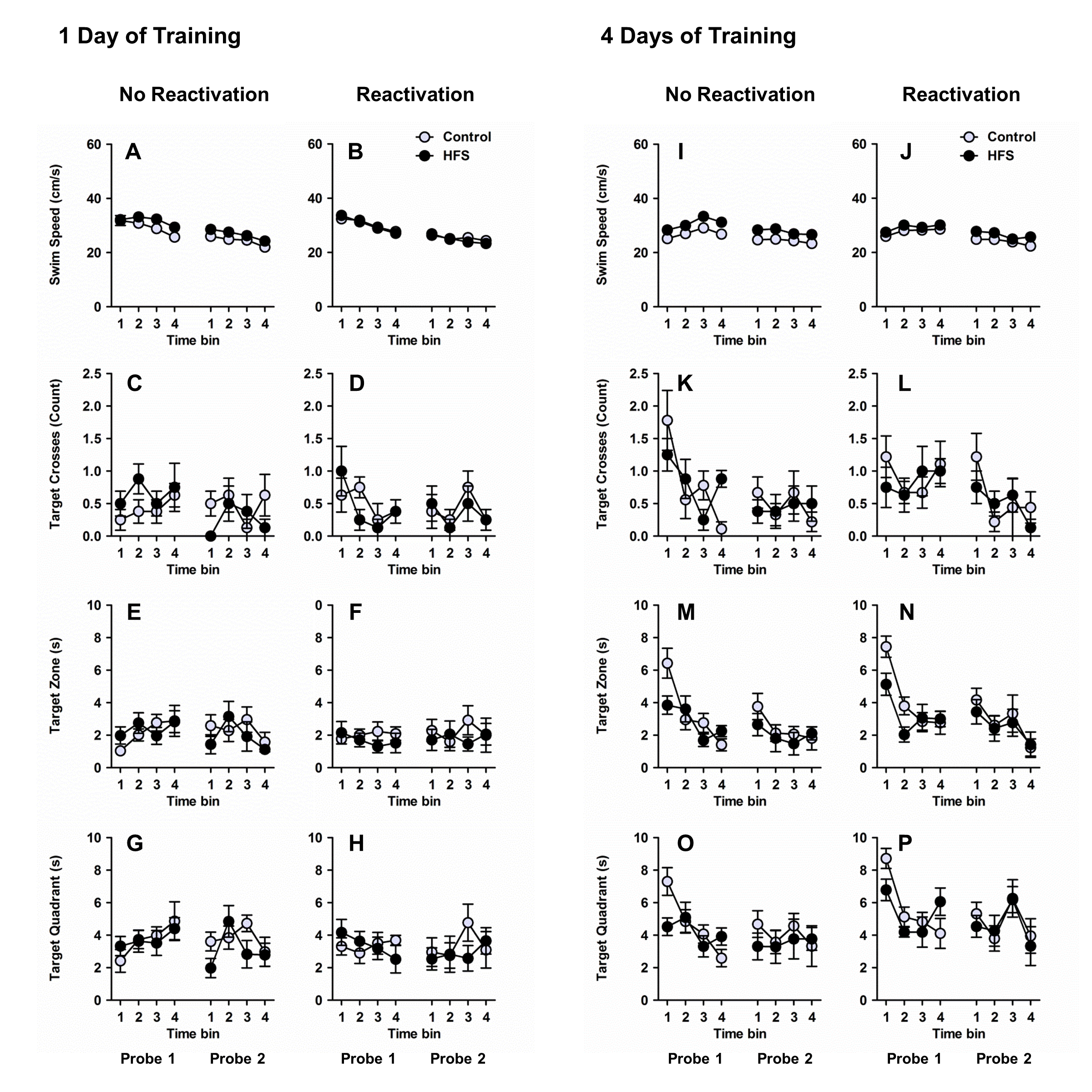

Supplement: Figure S1 — Time bin analysis of probe test performance. (ABIJ) Swim speed across 15-s time bins. (CDKL) Number of target crosses across 15-s time bins. (EFMN) Time spent in a 25-cm circular zone centered on the target location across 15-s time bins. (GHOP). Time spent in the target location quadrant across 15-s time bins. (TIF) [file pone.0100766.s001.tif]
