# Supplementary material for: Effect of High-Frequency Stimulation of the Perforant Path on Previously Acquired Spatial Memory in Rats: Influence of Memory Strength and Reactivation
Source: PLoS One. 2014 Jun 27;9(6):e100766. doi: 10.1371/journal.pone.0100766 (PMC4074056; doi:10.1371/journal.pone.0100766)
Supplement: Text S1 — Results of time bin analysis of probe test performance. (DOCX) [file pone.0100766.s002.docx]

**Text S1. Results of time bin analysis of probe test performance.**

To further test for differences in probe test performance among experimental conditions, we divided each 60-s probe test into four 15-s bins and performed ANOVAs with HFS, reactivation, and training as between-subject factors and probe and bin as within-subject factors. Significant interactions were followed by separate ANOVAs within specific factors of interest. This bin analysis was performed on swim speed and the three measures of persistence in searching for the platform (i.e., number of target location crosses, time in target zone, and time in target quadrant).

*Swim speed*. We found a significant training × probe × bin interaction (*F*_3,174_ = 12.61, *p* < .001), training × probe interaction (*F*_1,58_ = 9.58, *p* = .003), training × bin interaction (*F*_3,174_ = 16.53, *p* < .001), probe × bin interaction (*F*_3,174_ = 4.03, *p* = .008), probe main effect (*F*_1,58_ = 150.65, *p* < .001), and bin main effect (*F*_3,174_ = 15.93, *p* < .001) on swim speed (Figure S1ABIJ). We also found a significant reactivation × HFS interaction (*F*_1,58_ = 5.08, *p* = .028), indicating that HFS rats swam faster than control rats if memory for the platform was not reactivated prior to the probe tests (main effect of HFS within the no reactivation group collapsed across training group, *F*_1,31_ = 17.52, *p* < .001), whereas HFS and control rats showed equivalent swim speed if memory for the platform was reactivated prior to the probe tests (main effect of HFS within the reactivation group collapsed across training group, *p* > .05).

To test whether the effect of HFS on probe test performance among rats that received minimal (i.e., 1 day) training might be due to a disruption of procedural aspects of the task, we performed ANOVA with HFS and reactivation as between-subject factors, bin as a within-subject factor, and swim speed during the first probe test as the dependent measure. We found no significant interactions or main effects involving HFS (*p*s > .05; Figure S1AB).

*Persistence measures*. We found a significant training × HFS × reactivation × bin interaction on crosses (*F*_3,174_ = 2.82, *p* = .041), training × HFS × probe × bin interaction on time in quadrant (*F*_3,174_ = 2.89, *p* = .037), training × reactivation × probe × bin interaction on crosses (*F*_3,174_ = 4.22, *p* = .007) and time in quadrant (*F*_3,174_ = 2.82, *p* = .040), training × HFS × bin interaction on time in zone (*F*_3,174_ = 2.98, *p* = .033), training × probe × bin interaction on time in zone (*F*_3,174_ = 2.80, *p* = .041) and time in quadrant (*F*_3,174_ = 2.77, *p* = .043), training × reactivation interaction on time in zone (*F*_1,58_ = 5.22, *p* = .026) and time in quadrant (*F*_1,58_ = 9.49, *p* = .003), training × probe interaction on time in zone (*F*_1,58_ = 7.48, *p* = .008), training × bin interaction on all three dependent measures (crosses, *F*_3,174_ = 2.77, *p* = .043; time in zone, *F*_3,174_ = 15.91, *p* < .001; time in quadrant, *F*_3,174_ = 5.48, *p* = .001), probe × bin interaction on time in quadrant (*F*_3,174_ = 5.27, *p* = .002), training main effect on all three dependent measures (crosses, *F*_1,58_ = 17.87, *p* < .001; time in zone, *F*_1,58_ = 23.87, *p* < .001; time in quadrant, *F*_1,58_ = 30.90, *p* < .001), HFS main effect on time in zone (*F*_1,58_ = 4.90, *p* = .031), probe main effect on all three dependent measures (crosses, *F*_1,58_ = 18.17, *p* < .001; time in zone, *F*_1,58_ = 7.25, *p* = .009; time in quadrant, *F*_1,58_ = 6.25, *p* = .015), and bin main effect on crosses (*F*_3,174_ = 3.67, *p* = .013) and time in zone (*F*_3,174_ = 10.99, *p* < .001). To further analyze differences among experimental conditions, separate ANOVAs were performed within the 1-day and 4-day training groups.

Among rats that received 1 day of training, we found only a significant reactivation × bin interaction on crosses (*F*_3,174_ = 2.98, *p* = .036; Figure S1CD). Subsequent ANOVAs revealed no significant effects involving bin within the no reactivation group (Figure S1C, *p*s > .05) but a significant probe × bin interaction within the reactivation group (Figure S1D, *F*_3,174_ = 2.96, *p* = .043). There were no significant interactions or main effects for time in zone or time in quadrant (*p*s > .05).

Among rats that received 4 days of training, we found a significant probe × bin interaction on time in zone (*F*_1,58_ = 3.75, *p* = .014) and time in quadrant (*F*_1,58_ = 6.92, *p* < .001), probe main effect on all three dependent measures (crosses, *F*_1,58_ = 19.93, *p* < .001; time in zone, *F*_1,58_ = 17.11, *p* < .001; time in quadrant, *F*_1,58_ = 7.54, *p* = .010), and bin main effect on all three dependent measures (crosses, *F*_3,174_ = 4.98, *p* = .003; time in zone, *F*_3,174_ = 21.64, *p* < .001; time in quadrant, *F*_3,174_ = 5.56, *p* = .002).

Corroborating the findings reported in the main text, we also found a significant HFS × bin interaction (*F*_3,174_ = 3.01, *p* = .034) and HFS main effect on time in zone (*F*_1,58_ = 4.74, *p* = .038). Follow-up *t*-tests indicated that HFS rats spent less time in the target zone than control rats during the first 15-s time bin (collapsed across reactivation/no reactivation group and probe test, *t*_32_ = 2.83, *p* = .008; Figure S1MN), but there were no differences between HFS and control rats during subsequent time bins (*p*s > .05).

Finally, we found a significant reactivation × probe × bin on crosses (*F*_3,174_ = 3.21, *p* = .027). Follow-up *t*-tests indicated that rats in the reactivation group crossed the platform more often than rats in the no reactivation group during the fourth 15-s bin of the first probe test (collapsed across HFS/control group, *t*_32_ = 2.49, *p* = .018; Figure S1KL), but there were no differences between reactivation groups during the other time bins (*p*s > .05). Similar to the findings reported in the main text, we also found a significant reactivation main effect on time in zone (*F*_1,58_ = 4.69, *p* = .039; Figure S1MN) and time in quadrant (*F*_1,58_ = 12.27, *p* = .001; Figure S1OP), indicating that rats in the reactivation group spent more time in the target zone and quadrant compared to rats in the no reactivation group (collapsed across HFS/control group, probe, and bin).
